# Empowerment-based case management for treatment-resistant schizophrenia: a three-year single case report

**DOI:** 10.3389/fpsyt.2026.1760312

**Published:** 2026-02-09

**Authors:** Cong Wang, Xianbin Li, Jinsong Chen, Xiaohong Li, Wenpeng Hou, Ning Dong

**Affiliations:** 1 Beijing Key Laboratory of Mental Disorders, National Clinical Research Center for Mental Disorders & National Center for Mental Disorders, Beijing Anding Hospital, Capital Medical University, Beijing, China; 2 Advanced Innovation Center for Human Brain Protection, Beijing, China; 3 Center for Studies of Sociological Theory & Method, Department of Social Work, School of Social Research, Renmin University of China, Beijing, China; 4 Department of Psychiatry, Beijing Huilongguan Hospital Affiliated Capital Medical University, Beijing, China; 5 Peking University, Huilonguan Clinical Medical School Beijing, Beijing, China

**Keywords:** case management, empowerment theory, rehabilitation, schizophrenia, treatment-resistant

## Abstract

This case report describes the implementation and observed outcomes of an empowerment-oriented case management approach in a patient with treatment-resistant schizophrenia (TRS), addressing the limitations of conventional approaches that often neglect patient autonomy and subjective agency. Through the implementation of a multidisciplinary, four-dimensional empowerment framework—comprising pathological treatment, psychological cognition, social engagement, and family care—a 34-year-old male TRS patient received targeted intervention over a three-year period. Quantitative assessments, including PANSS, HRSD, SDSS, CGI, MRSS, and FAES II-CV, demonstrated substantial improvements: PANSS total score decreased from 90 to 61, CGI severity score reduced from 6 to 3, and MRSS dependency subscale declined from 29 to 14. Qualitative interviews further revealed enhanced medication adherence, symptom self-monitoring, and social functioning, alongside increased daily autonomy, reduced stigma, and improved family dynamics. These preliminary observations from a single case suggest potential benefits that warrant systematic investigation through controlled studies. The temporal associations observed between the intervention and improvements cannot establish causality but provide hypotheses for future research.

## 1 Introduction

Schizophrenia is a severe mental disorder with unclear causes, characterized by severe cognitive, thought, emotional, and behavioral dysfunctions, which results in diminished occupational and social functioning (1). Many patients are diagnosed with Treatment-Resistant Schizophrenia (TRS), despite receiving appropriate dosages and treatment regimens involving two or more antipsychotic medications with distinct chemical structures, fail to achieve substantial therapeutic outcomes, and persist in experiencing both positive and negative symptoms (2). In response to this issue, existing research suggests that case management is highly effective in improving the psychotic symptoms of TRS, reducing relapse and disability rates, and enhancing quality of life (3).

Case management models are broadly categorized into three types based on service orientation: the Strengths Model emphasizing personal and environmental assets to foster recovery (4), the Expanded Broker Model linking patients to rehabilitation systems via resource evaluation (5), and the Rehabilitation Model targeting functional deficits to establish service goals. These goal-driven frameworks priorities identifying patients’ strengths and capabilities to unlock their potential. Clinically, case management is further divided into inpatient and community-based approaches. The former employs the Clinical Model during acute phases, focusing on symptom control, treatment adherence, and self-management to facilitate discharge (6). The latter adopts multidisciplinary teamwork to deliver sustained community care (7). However, current practices often inadequately address individualized rehabilitation, particularly in fostering independence. For treatment-resistant schizophrenia (TRS), biological and social impairments synergistically hinder normative role fulfilment. Transitioning care from hospitals to communities introduces instability, as community diagnoses frequently rely on incomplete historical records, while patients face heightened stigma, isolation, and diminished self-efficacy (8). Bridging these gaps requires innovative strategies to enhance self-development and secure compensatory social support (9). Given the innovative nature of this integrated empowerment approach and the complexity of TRS, we present this detailed single-case report to: (1) demonstrate feasibility of implementation within a multidisciplinary rehabilitation setting, (2) document comprehensive mixed-methods assessment protocols combining standardized scales with longitudinal qualitative interviews, and (3) generate specific hypotheses for future controlled studies. This approach aligns with established intervention development frameworks where detailed case analysis provides essential preliminary evidence before definitive efficacy trials.

## 2 Background

Empowerment theory, rooted in social work, emphasizes individual-social system interactions to enhance well-being through rights restoration (10). Studies in community mental health settings demonstrate empowerment’s role in recovery via resource access, community engagement, and treatment autonomy (11, 12). Empirical evidence frames empowerment as integrating individual strengths with systemic policies to drive social change (13). Nevertheless, macro welfare initiatives often neglect individual capacity-building. Unlike public welfare and policy advocacy, a collaborative focus on individual ability-building should be prioritized to enhance patients’ capacities and empower them to take charge of self-care and decision-making.

Starting from the individual, medical empowerment enhances autonomy in health management by improving treatment adherence and stabilizing conditions through patient education on disease mechanisms (14). Psychological empowerment fosters self-awareness and intrinsic motivation, overcoming cognitive barriers to independent action (15). Social empowerment rebuilds functional capacities via daily skill training, enabling adaptive navigation of environmental challenges and resource mobilization (16). Collectively, within the bio-psycho-social framework, these strategies synergies to cultivate self-reliant illness management and preserve autonomy in coexisting with chronic disease.

This study is predicated on the idea that people with mental illness can develop their ability for cognitive renewal and self-remodeling. Taking a case of TRS in the day rehabilitation center of Beijing Anding Hospital as an example, case management guided by the empowerment theory is implemented for the patient. The recovery status is evaluated using both quantitative research and qualitative assessment, aiming to identify strategies for enhancing the prognosis of TRS and propose thoughts for improving the prognosis of TRS.

## 3 Method

### 3.1 Ethical considerations and study design

This case report was conducted in accordance with the Declaration of Helsinki and approved by the Ethics Committee of Beijing Anding Hospital. Written informed consent was obtained from the patient for both participation in the rehabilitation program and publication of this case report, including clinical data and treatment outcomes. The report follows the CARE (CAse REport) guidelines to ensure completeness and transparency in reporting. Qualitative data collection and analysis followed established protocols for rigor. Semi-structured interviews were conducted at 3-month intervals throughout the 3-year follow-up period, totaling 12 formal interviews supplemented by informal conversations during clinical encounters. All interviews were audio-recorded, transcribed verbatim in Mandarin, and analyzed using thematic analysis. The analytical process involved iterative coding by two independent researchers, with themes developed both inductively from the data and deductively from the empowerment framework. Trustworthiness was established through triangulation of data sources (patient interviews, clinical observations, family reports, and team meeting notes), member checking where the patient reviewed and validated our interpretations at 6-month intervals, and maintenance of an audit trail documenting all analytical decisions. The prolonged 3-year engagement period allowed for deep understanding of the patient’s recovery trajectory and verification of observed changes over time.

### 3.2 Presenting problem and client description

The patient is a 34-year-old man with schizophrenia, multiple episodes, currently in partial remission (DSM-5 295.90), presenting with a 20-year illness course. Illness onset occurred at age 14 during early adolescence, disrupting his education after completing 9th grade (junior high school). He never achieved employment due to continuous symptoms throughout young adulthood. Prior to case management enrollment at age 32, he had experienced approximately 18 years of persistent positive symptoms (auditory hallucinations, paranoid delusions) and negative symptoms (avolition, social withdrawal) with seven psychiatric hospitalizations across three Beijing hospitals for acute exacerbations. Previous interventions included standard inpatient pharmacotherapy and brief psychoeducation, but no systematic psychosocial rehabilitation or structured case management. At baseline, he lived alone in an apartment with his father living separately but providing regular financial support and periodic visits. The patient had no significant medical comorbidities, though he presented with severe depressive symptoms (baseline HAMD = 30) and stigma-related anxiety alongside his psychotic symptoms.

Treatment resistance was systematically confirmed according to international consensus criteria: the patient had failed adequate trials of risperidone (titrated to 6mg/day for 8 weeks with plasma levels confirming adherence), olanzapine (20mg/day for 12 weeks), and aripiprazole (30mg/day for 10 weeks) before initiating clozapine. The patient had been maintained on a stable medication regimen (clozapine 400mg/day, aripiprazole 10mg/day, sertraline 50mg/day) for 18 months prior to case management enrollment, with therapeutic clozapine levels (mean 425 ± 75 ng/mL, monitored monthly) and no medication adjustments throughout the entire 3-year intervention period. Despite this optimized and stable pharmacological treatment, the patient continued to experience residual symptoms with significant functional impairment (baseline PANSS = 90, SDSS = 32), indicating that he had plateaued in pharmacological response before psychosocial intervention began. His treatment history was verified through medical records from three psychiatric hospitals in Beijing, pharmacy dispensing records, and plasma level monitoring data. The presence of treatment resistance despite optimal pharmacological management, combined with substantial family history of schizophrenia (mother, maternal grandfather, and uncle affected, with maternal suicide during acute psychosis), provided the rationale for implementing an intensive psychosocial intervention approach.

### 3.3 Service model

**Figure 1:**
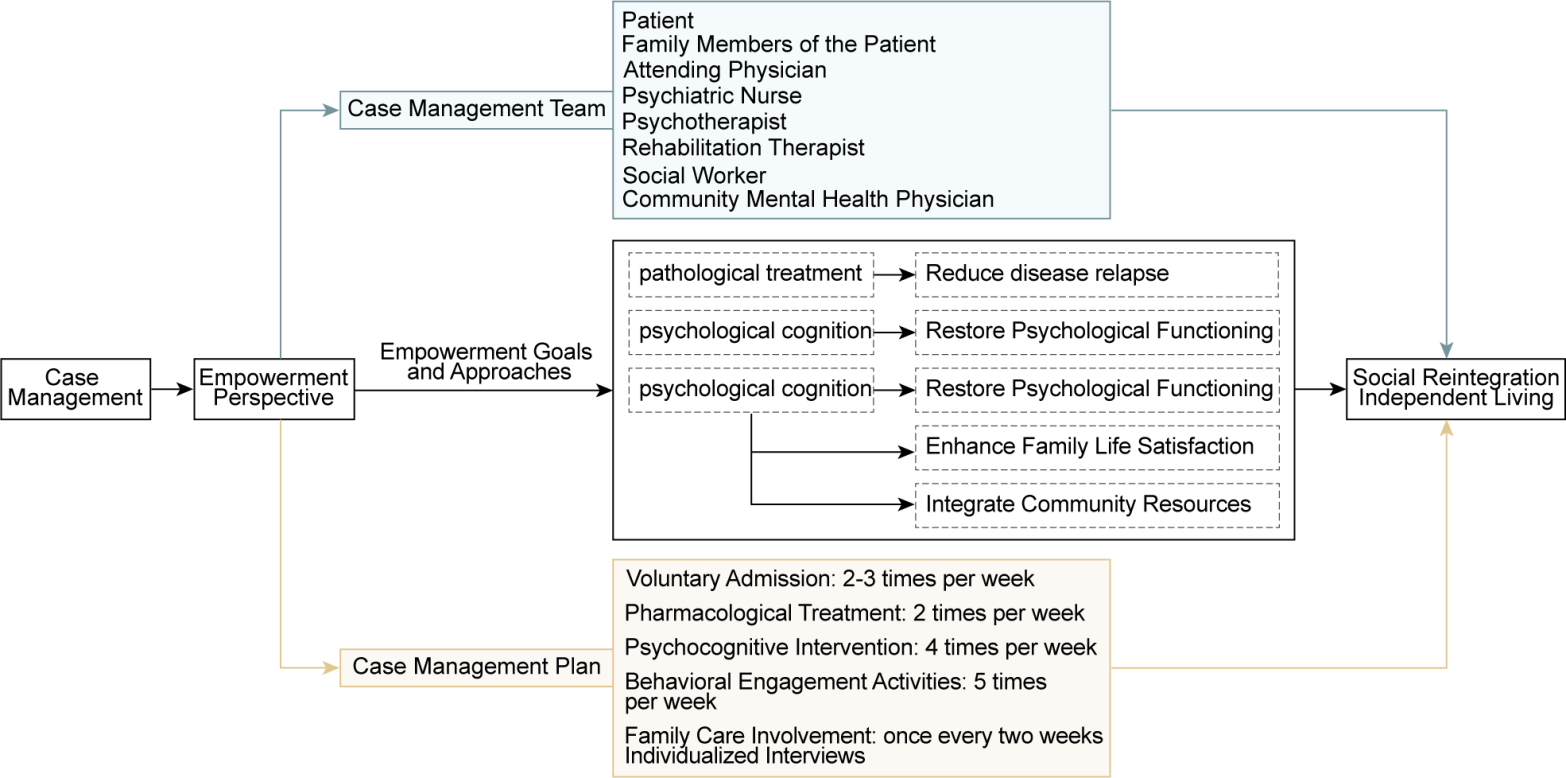
The case management model.

Case management is a comprehensive intervention program that provides patients with personalized medical, psychological, and social support through multidisciplinary collaboration to facilitate their ability in daily life and smooth integration into the social environment (**Figure 1**). Case management consists of three aspects: the service model, case management team and plan. Below is a detailed discussion of these.

Based on individual capacity building, the empowerment perspective aims to discuss risk-compensating factors from the relationships between health and illness, ability and disability, and strengths and weaknesses in individuals and their surroundings to construct an empowerment framework that integrates pathological treatment, psychological cognition, and social interaction. Additionally, this study categorizes social interaction empowerment into two dimensions: empowerment through involvement with others in the hospital environment and the extended system outside the hospital, which includes community and family, incorporating the living circumstances of individuals in recovery. This study highlights the transition from inpatient treatment to home life, unlike previous models concentrated solely on hospital or communal treatment. It encourages applying rehabilitation knowledge gained in hospitals to address difficulties faced in daily life, promoting reflection on rehabilitation experiences within ordinary situations, and facilitating feedback to the case team. This enables prompt revisions to the rehabilitation plan, resulting in a continuous range of care during the acute episodes, stable periods, and maintenance phases of mental illness.

#### 3.3.1 Empowerment for pathological treatment

Rehabilitation for TRS should focus on pharmacological interventions, accompanied by consistent monitoring of blood drug levels and potential adverse drug reactions. This requires patients to possess certain autonomy in managing medication and symptoms. Therefore, educating patients on their ailments is essential for pathological treatment. This stage aims to foster adequate medication adherence and enhance disease comprehension, which is crucial for sustaining stability and preventing relapse during rehabilitation (17). Pathological treatment empowerment employs a systematic approach to medication management and symptom control in the medical field. Through disease education, patients are guided to attain self-sustenance through ongoing training, actively engaging in the decision-making process regarding their treatment and rehabilitation management.

*Clinical Vignette 1 (Medication Negotiation):* At 8 months, the patient reported: ‘The aripiprazole makes me restless; I can’t sit still. I want to stop it.’ Rather than directive refusal, the psychiatrist explored: ‘The restlessness is difficult. What bothers you most—physical discomfort or worry about appearance?’ Patient: ‘Both. I look strange moving around.’ Psychiatrist: ‘Let’s problem-solve together. We could reduce the dose to 5mg as a trial, or switch timing to bedtime. Which would you prefer trying first?’ Patient chose dose reduction. After 2 weeks with continued monitoring, the patient reported reduced restlessness without symptom worsening, agreeing to maintain the adjustment. This negotiation operationalized shared decision-making while maintaining clinical safety.

#### 3.3.2 Empowerment for psychological cognition

Psychotherapy and cognitive training are critical methods for promoting social reintegration. TRS confronts stigma, anxiety, and depression generated by frequently enduring psychotic symptoms, including hallucinations and delusions, often alongside manifestations such as diminished willpower and emotional blunting. Empowerment for psychological cognition tries to address these adverse conditions through artistic expression and other traditional psychological interventions. This process facilitates the gradual restoration of insight into illness while also contributing to the development of self-expression and self-awareness, the reactivation of imagination, and the enhancement of psychological resilience. All of this aids the patients in regaining hope and motivation for recovery, strengthening their subjectivity and motivation for life (18).

#### 3.3.3 Empowerment for social participation

This section encompasses the individual’s engagement in activities, familial care, and community welfare. There is a reciprocal relationship between activity and positive psychology. Thus, the initiative might be effectively increased by strengthening psychological processes, modifying cognitive structures, and improving social functioning. The empowerment process designed for active participation proceeds as follows: enhancing personal self-care abilities, improving interpersonal expression and communication skills, increasing strategies to tackle complex situations, and developing labor expertise to obtain survival resources. This empowerment approach aims to modify patients’ cognitive and coping strategies regarding external individuals and events through rehabilitative social skills training, promoting positive interaction patterns.

The continuous rehabilitation process of patients is greatly aided by their families and caregivers, who are primarily responsible for supplying care. Home care empowerment relies on family interventions to improve the professional skills of caregivers throughout various fields, including emergency response, patient care, and illness management. After reducing the physical and psychological stress experienced by caregivers, the ultimate goal is to enhance the patient’s quality of life, elevate satisfaction levels for both the patient and their family regarding life experiences, and promote a professional and harmonious rehabilitation environment (19).

The community is vital for delivering social welfare support related to employment, medical care, and social interactions as the second central system in which the patient resides. Thus, activating social welfare benefits and reintegration into the community are directly related to in-hospital rehabilitation training. With social work services, the recuperation procedure associated with community welfare empowerment considers the patient’s awareness and accessibility of supportive resources in the community. This lays the groundwork for long-term growth and social integration by assisting patients in identifying and integrating possible resources.

*Clinical Vignette 2 (Goal-Setting Session):* At 12 months, the rehabilitation therapist initiated goal-setting: ‘What would meaningful progress look like for you in the next 3 months?’ Patient: ‘I want to work, but I’m scared.’ Therapist: ‘What specifically worries you?’ Patient: ‘Making mistakes, people seeing I’m different.’ Therapist: ‘Let’s break this down. What’s one small step toward work that feels manageable?’ Patient: ‘Maybe volunteering here first?’ The team collaboratively designed a volunteer role (newspaper distribution) matching his readiness level, with explicit agreement that he could pause if overwhelmed. This process honored patient agency while providing scaffolded support, exemplifying empowerment through graduated autonomy rather than premature job placement.

#### 3.3.4 Operationalizing empowerment and shared decision-making

Empowerment techniques were operationalized through structured protocols in each dimension. In pathological treatment, shared decision-making involved presenting medication options with risk-benefit profiles, asking “Which side effect concerns you most?”, and collaboratively adjusting timing (e.g., switching evening doses to reduce daytime sedation per patient preference). For psychological interventions, the patient selected art therapy activities from available options and determined disclosure depth in group sessions. Social participation training used graduated autonomy: staff initially accompanied community outings, then transitioned to patient-led activities with available support.

Balancing autonomy with clinical risk followed a tiered protocol: (1) Low-risk decisions (activity choices, daily schedule) were fully patient-directed; (2) Medium-risk decisions (medication timing adjustments, community outing destinations) involved collaborative discussion with documentation of patient preferences and clinical reasoning; (3) High-risk situations (acute symptom exacerbation, medication discontinuation requests) triggered team consultation, with the psychiatrist explaining clinical concerns while exploring patient’s underlying needs (e.g., side effect intolerance prompting discontinuation request led to medication adjustment rather than override). One example occurred at 8 months when the patient wanted to stop aripiprazole due to restlessness. Rather than refusing, the team explored alternatives, reduced the dose from 10mg to 5mg as a trial, and monitored outcomes collaboratively—the patient agreed to maintain the lower dose after experiencing reduced restlessness without symptom worsening.

### 3.4 Case management team

Multidisciplinary case management is conducted by a psychiatrist, psychiatric nurse, psychotherapist, social worker, rehabilitation therapist, and community mental health physician. The psychiatrist monitors medical, psychological, and social rehabilitation as the principal case manager. The psychiatrist also collaborates closely and holds monthly meetings to assess patient progress and modify therapeutic strategies. Additional team members, including the nurse, psychotherapist, and rehabilitation therapist, must execute daily rehabilitation training and track patient development. The social worker conducts weekly case meetings to understand the patients and their families’ conditions. Based on this knowledge, the social worker actively integrates resources to establish a continual rehabilitation system that thrives inside and outside the hospital, promoting contact and collaboration between the medical facility and the community. With an emphasis on interprofessional collaboration and patient-centered care, the multidisciplinary team replaces the conventional single-line treatment model with a collaborative, dynamic approach that relies on shared decision-making. Empowerment theory is reflected in the patient-team relationship throughout this process and integrated into the patient’s treatment and rehabilitation.

Team coordination operated through defined roles and communication structures. The principal case manager (psychiatrist) synthesized input from all team members, made final clinical decisions, and maintained treatment plan coherence. Weekly case meetings followed a structured format: (1) each professional reported observations from their domain (nurse: daily functioning; therapist: session engagement; social worker: family/community context), (2) discrepancies were discussed openly (e.g., when the rehabilitation therapist observed motivation decline that the psychiatrist had not detected), and (3) the principal case manager integrated perspectives into adjusted care plans. Disagreements were resolved through evidence presentation and return to patient goals—for instance, when the social worker advocated for earlier employment placement while the psychiatrist recommended continued skill-building, the patient’s stated priority (reducing social anxiety before job-seeking) guided the decision to delay employment support for 3 months. This collaborative model prevented fragmentation while preserving professional autonomy within domains.

### 3.5 Operational plan

The patient voluntarily admits to hospital 2–3 times weekly to engage in rehabilitation courses. In a week, pathological treatment is once, the psychological, cognitive course is 2 times, and the action participation course occurs 3–4 times. The family care course involved the patient’s father once every two weeks. The primary trainers of these courses included psychiatrists, psychiatric rehabilitation therapists, and psychotherapists. In addition, community welfare empowerment is implemented through biweekly to monthly case meetings between social workers and patients, alongside consistent communication with the patients’ families and their communities. As of February 2025, this patient has engaged in case management for three years.

### 3.6 Assessment method

A mixed-methods approach that combines qualitative interviews and quantitative data is utilized to evaluate the efficacy of the TRS case management and comprehensively assess patients’ recovery status based on an empowerment theory.

Quantitative assessment is conducted at various time intervals utilizing the Positive and Negative Syndrome Scale (PANSS), Hamilton Depression Scale (HAMD), Social Disability Screening Schedule (SDSS), Clinical Global Impression (CGI), Morningside Rehabilitation Status Scale (MRSS), and the Chinese Version of Family Adaptability and Cohesion Evaluation Scale II(FAES II-CV). This evaluation considers clinical symptoms, psychological functioning, social adaptability, and familial environment. All quantitative assessments were administered by the treating psychiatrist who served as the primary case manager, without blinding to treatment status or intervention progress.

The qualitative interview combines empowerment theory and assessment scales to refine interview themes, using a semi-structured interview format that focuses on changes in the patient’s self-management of the illness, expressive and emotional states, social interaction skills, changes in the family environment, and community support contacts. Given the intricacy and unpredictability of TRS, this interview format is adaptable.

### 3.7 Qualitative analysis procedure

Thematic analysis followed Braun and Clarke’s six-phase approach. Interview audio recordings were transcribed verbatim in Mandarin. Two researchers (ND, CW) independently conducted initial coding using NVivo 12 software. The coding process involved: (1) familiarization through repeated reading, (2) generating initial codes, (3) searching for themes by grouping codes, (4) reviewing themes against transcripts, (5) defining and naming themes, and (6) selecting representative quotes. Coders met biweekly to compare coding, discuss discrepancies, and reach consensus through discussion and reference to the empowerment framework. Inter-coder agreement was calculated at 87% before consensus discussions. Final themes were member-checked with the patient at 18, 24, and 30 months to ensure interpretive accuracy.

## 4 Findings

### 4.1 Quantitative assessment results

Quantitative assessments were conducted using standardized rating scales at six key time points—baseline (upon patient enrollment in case management), 1 month, 3 months, 12 months, 24 months, and 33 months of rehabilitation. The results are detailed in **Table 1**.

**Table 1:** Scale results of the case during treatment.

Scales	Subscales	Baseline	1st	3rd	12th	24th	33th
CGI	CGI-S	6	5	5	4	4	3
	CGI-G	0	4	5	2	2	1
	CGI-E	00	14	14	10	06	02
PANSS	PANSS-P	22	21	20	19	14	12
	PANSS-N	24	23	23	22	17	17
	PANSS-G	44	40	40	38	30	32
	PANSS-T	90	84	83	79	61	61
HAMD	Total Score	30	28	22	35	25	18
FAES II-CV	D-C	8	8	7	7	10	12
	D-A	10	11	10	10	5	4
SDSS	Total Score	32	32	30	28	25	25
MRSS	Total Score	124	120	110	104	68	58
	D	29	28	25	25	17	14
	IOL	29	25	22	22	18	14
	SI	42	40	40	35	20	19
	CSDB	24	23	23	22	13	11

Following the intervention of Treatment-Resistant Schizophrenia (TRS) case management, the Clinical Global Impression-Severity (CGI-S) score decreased from 6 to 3, and the Clinical Global Impression-Improvement (CGI-I) score improved from 4 at 1 month of rehabilitation to 1. The efficacy index also showed marked improvement, decreasing from 14 at 1 month to 2.

The Positive and Negative Syndrome Scale (PANSS) total score was reduced from 90 to 61, with the positive symptom subscale decreasing from 22 to 12, the negative symptom subscale from 24 to 17, and the general psychopathology subscale from 44 to 32. PANSS and CGI primarily assess the effectiveness of disease interventions and clinical symptoms. The findings indicate significant improvements in patients’ conditions, symptoms, and treatment efficacy as TRS case management progressed.

The Hamilton Depression Scale (HAMD) score initially increased from 30 to 35 due to heightened illness-related stigma but subsequently decreased to 18 as treatment advanced. HAMD primarily evaluates patients’ emotional states, suggesting that depressive symptoms were more pronounced in the mid-stage of treatment but significantly alleviated by the end of the intervention.

Regarding family dynamics, the dissatisfaction with intimacy score on the Family Adaptability and Cohesion Evaluation Scale II-Chinese Version (FAES II-CV) increased from 8 to 12. In contrast, dissatisfaction with adaptability decreased from 10 to 4. FAES II-CV assesses family intimacy and adaptability, and the results indicate a gradual improvement in dissatisfaction with adaptability. However, as treatment progressed and symptoms improved, patients expressed increased dissatisfaction with family intimacy.

The Social Disability Screening Schedule (SDSS) total score decreased from 32 to 25, while the Modified Rehabilitation Social Scale (MRSS) total score improved from 124 to 58. The dependency subscale decreased from 29 to 14, the lack of activity subscale from 29 to 14, the social interaction subscale from 42 to 19, and the current symptoms and abnormal behaviors subscale from 24 to 11. SDSS and MRSS primarily assess rehabilitation status, and both scales demonstrated significant improvements following case management interventions. Notably, the MRSS social interaction subscale showed substantial enhancement, indicating marked improvements in patients’ social functioning.

Overall, the frequency of auditory hallucinations and delusions decreased, affective responses became more enriched, social functioning significantly improved, and relationships with family members became more harmonious.

#### 4.1.1 Recovery from illness and improvement in social functioning

Visual analysis of the longitudinal data revealed parallel trends between clinical improvement and functional outcomes. As CGI-S scores decreased from 6 to 3 (50% reduction), SDSS scores similarly decreased from 32 to 25 (21.9% reduction). The PANSS total score reduction from 90 to 61 was accompanied by improvements in MRSS scores from 124 to 58. These concurrent improvements across multiple domains suggest a comprehensive therapeutic response pattern, though causality cannot be inferred from this single case observation. These concurrent improvements across multiple domains suggest a comprehensive therapeutic response pattern, though causality cannot be inferred from this single case observation.

#### 4.1.2 Rehabilitation function and improvement in family function

In this study, we utilized the Family Adaptability and Cohesion Evaluation Scale II-Chinese Version (FAES II-CV) to assess family function, with a primary focus on adaptability and cohesion—two core dimensions of family function that reflect the family’s ability to respond to external changes and the emotional bonds among family members, respectively.

Our findings indicate that during individualized intervention, the scores of the Dependency, Socialization, Lack of Activity, Symptoms, and Aberrant Behavior subscales of the Multidimensional Recovery Support Scale (MRSS) showed significant reductions. This suggests that multifaceted interventions occurred alongside improvement of rehabilitation function in patients.

It is noteworthy that an increase in the dissatisfaction score of the FAES II-CV cohesion dimension often implies that family members feel dissatisfied with emotional connections, support, and a sense of belonging within the family. This dissatisfaction may stem from emotional detachment, increased conflict, or a diminished sense of belonging. However, in the case of patients with schizophrenia, later-stage interviews revealed an emerging need for emotional support from the family. Expressions such as “I feel lonely and wish I had someone at home to accompany me” suggest an enriched emotional experience and are associated with an improvement in negative symptoms.

**Figure 2:**
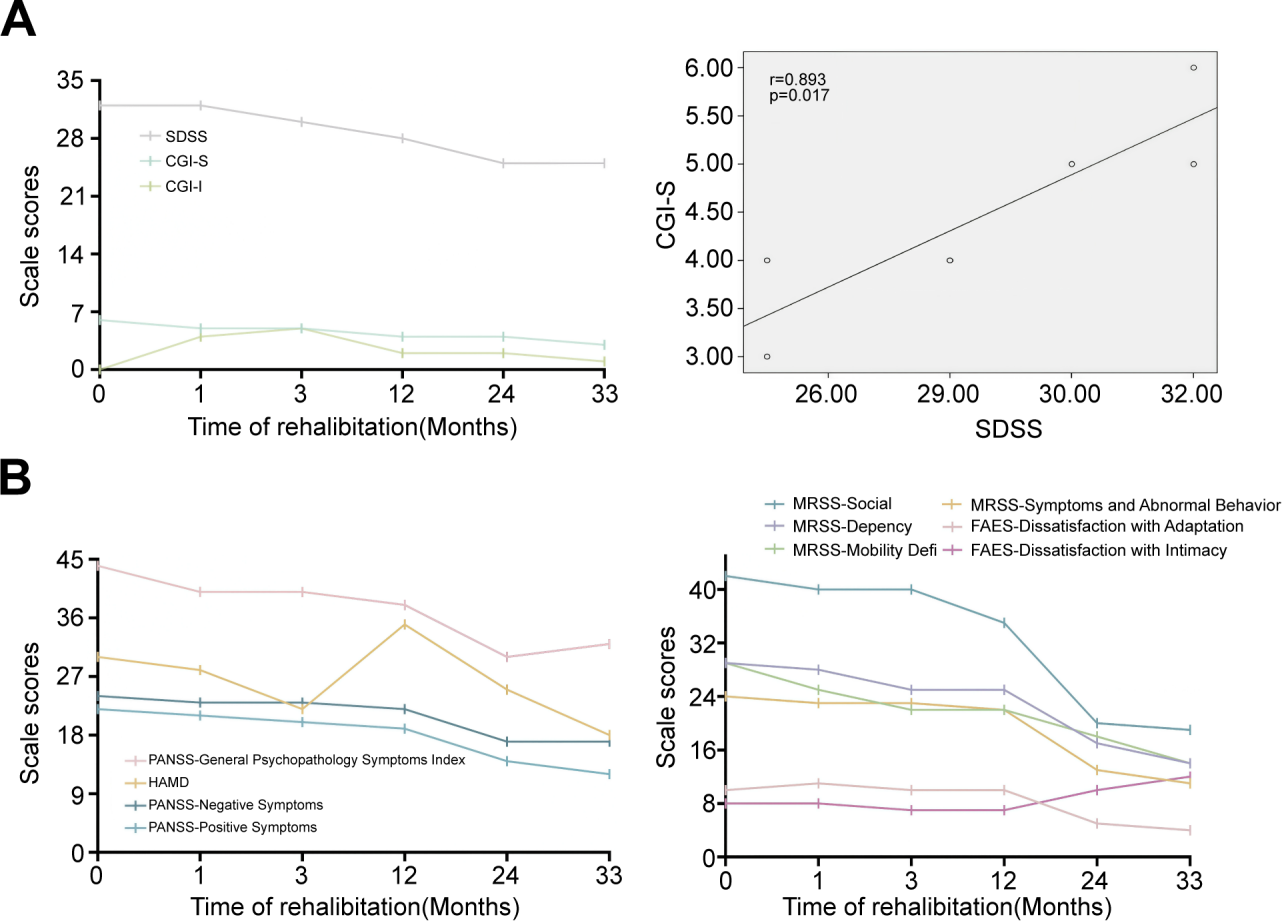
Clinical rating scale outcomes.

Furthermore, due to the integration of family intervention program during the intervention period, the dissatisfaction score of the FAES II-CV adaptability dimension decreased. This indicates that patients became more satisfied with their family’s ability to cope with change, potentially reflecting improvements in problem-solving skills, more flexible role distribution, and enhanced communication and collaboration. These observations indicate an overall improvement in family function, as illustrated in **Figure 2A**.

#### 4.1.3 Trends in symptom improvement and emotional changes in psychiatric disorders

The three subscales of the Positive and Negative Syndrome Scale (PANSS)—positive symptoms, negative symptoms, and general psychopathology—showed varying degrees of improvement (**Figure 2B**). Notably, after the patient’s condition stabilized, the Hamilton Depression Rating Scale (HAMD) indicated fluctuations in depressive mood. Interviews conducted by social workers revealed that these mood fluctuations were closely associated with the patient’s sense of stigma. This abnormality was promptly detected through case management, allowing for timely adjustments in treatment strategies and targeted interventions. Subsequently, the patient’s depressive symptoms improved in the later stages.

#### 4.1.4 Integration of quantitative and qualitative findings

Quantitative improvements were meaningfully reflected in the patient’s subjective narrative. The PANSS negative symptom reduction (24→17) corresponded with his emotional enrichment descriptions: from ‘I don’t feel anything’ at baseline to ‘I feel lonely and want companionship’ at 24 months. The MRSS social interaction improvement (42→19) aligned with his behavioral shift from mask-wearing and gaze avoidance to initiating eye contact and seeking connections. The HAMD fluctuation at 12 months (rising to 35) was explained through his qualitative accounts of heightened stigma awareness, demonstrating how mixed methods revealed recovery nuances that quantitative data alone would miss.

### 4.2 Qualitative assessment results

Trustworthiness of Qualitative Findings To ensure rigor in qualitative analysis, we employed multiple strategies: (1) prolonged engagement through 3-year follow-up; (2) triangulation of data sources (patient interviews, clinical observations, family reports); (3) member checking of interpreted themes; (4) maintenance of an audit trail documenting analytical decisions. Patient quotes presented below were originally in Mandarin and have been translated with back-translation verification. Some represent condensed summaries approved by the patient rather than verbatim statements. Thematic analysis identified five core themes aligned with the empowerment framework dimensions (**Table 2**).

**Table 2:** Qualitative themes and empowerment framework.

Theme	Key transformation	Timeline	Empowerment dimension
From Passive Compliance to Medication Ownership	Developing autonomous symptom monitoring and medication management	0–36 months (foundational)	Pathological treatment
Emotional Reawakening and Stigma Reframing	Restoring affective capacity and reconstructing social identity	6–36 months (critical shift 12–24 months)	Psychological cognition
Reclaiming Social Roles and Community Presence	Progressing from isolation to active social participation	3–36 months (progressive)	Social participation
Reconciliation and Relational Repair	Transforming paternal relationship through understanding	12–36 months (gradual deepening)	Family care
From Concealment to Strategic Community Engagement	Shifting from stigma avoidance to resource advocacy	18–36 months (later emergence)	Community welfare

#### 4.2.1 From passive compliance to medication ownership

The patient’s ability to self-manage medication and monitor symptoms has significantly improved. In his resettled community, he has shown symptoms in public multiple times since developing the illness in adolescence. Fear surrounding stigma spurred an intense desire for symptom stability, which then formed a cognitive cycle of “medication—symptom relief—feeling better—maintaining normalcy” that strengthened medication adherence. Nevertheless, the long-term effects of schizophrenia have impaired his brain function, leading to a hazy comprehension of basic pharmaceutical information like names, side effects, and dosage regimens he used. Through repeated training, the patient has dramatically improved drug safety by learning to distinguish between various medications based on their size, shape, and color.

Furthermore, Symptom monitoring training has significantly enhanced the individual’s self-awareness regarding symptoms and relapses, allowing proactive communication with healthcare providers and feedback on changes. The patient has reported to social workers in several interviews experiences of perceptual disturbances about the real world and cognitive delusions of reference and persecution. For instance, he remarked, “I encountered my previous primary physician at the hospital, but he is from a different facility—how is he present here?” and “I met a fellow patient at the basketball court; we were previously hospitalized together at another facility”. The patient frequently questioned the veracity of these impressions, saying “I need to check,” or “I am not sure if I am seeing or hearing things because my illness is getting worse (relapsing).” Those actions illustrate patient perceives an increased vigilance regarding symptoms and relapses relative to previous experiences. Early interviews revealed limited medication understanding: ‘I don’t know what these pills do; I just take them because the doctor said so.’ After six months of training, he demonstrated active engagement: ‘Now I can tell the white pill from the pink one. If I forget medication and hear voices getting louder at night, I take it immediately.’ At 24 months, he articulated deeper insight: ‘Medication doesn’t cure me, but helps me control symptoms. I need to learn to live with the illness, not wait to be fixed. His symptom awareness evolved from passive confusion to active monitoring. Early interview (3 months): “I saw my old doctor here—why? Am I seeing things?” Later (27 months): “When voices get louder after missing medication, I know it’s a warning. I take the pill and tell the doctor.

#### 4.2.2 Emotional reawakening and stigma reframing

The patient suffers from loneliness due to earlier illness onset, lack of family support, and communal isolation. His capacity to respond to outside issues is further impeded by emotional and behavioral disruptions caused by schizophrenia. Psychotherapy sessions are conducted to enhance his awareness and exploration of emotions and improve his ability for self-expression, so he gradually expresses his feelings and requirements using straightforward statements, such as “I feel lonely” or “I want to make friends.” His loneliness may not always be positive, but it demonstrates diverse emotional experiences, which might help him recover. The external indicators represent an upbeat reaction to the patient for interaction with others and better psychological functioning during the case management process.

Additionally, the patient gradually has a greater comprehension of the stigma that emerges from community gossip and rejection linked to himself and his family’s schizophrenia diagnosis. The patient initially steered deep discussions about stigma by stating, “Neighbors are not good people,” attributing his “disgust and hatred” triggered by their rejection and indifference to his need to look strong and keep face. As the rehabilitation training altered his cognition by enabling systematic monitoring and exploring his thoughts, the patient was equipped with a more rational comprehension of the nature and causality of his experiences. Over time, in response to unfavorable feedback from others, the patient altered his thinking, arguing, “I simply ignore them and concentrate on taking care of myself.” He also opened up to social workers and doctors. The patient started to open up to social workers and doctors, “My behavior when I was sick scared others,” and “Neighbors were afraid of me, but now I’m better, and I can try to make friends with them.” Upon recognizing the negative perceptions generated by the illness in public contexts, the patient adopted a more objective stance towards the stigma. His cognitive transformation regarding stigma evolved gradually. Initially: ‘Neighbors are bad people who discriminate against my family.’ At 12 months, he began questioning: ‘Maybe my behavior when sick frightened them?’ By 24 months, he demonstrated mature perspective: ‘I did strange things when ill—their fear was understandable. Now I’m stable and can rebuild relationships slowly. I don’t need everyone to understand me, but I can choose to befriend those who are kind.

#### 4.2.3 Reclaiming social roles and community presence

Active participation is used to train for adaptation to social contexts. Patient self-reports, team observations, and social worker interviews all indicated that daily life skills training, which addresses fundamental survival needs like food, clothes, housing, and transportation, significantly improved the patient’s ability to perform daily tasks, uphold personal hygiene, dress suitably, and follow a regular daily schedule. For instance, the patient could buy bread and buns independently and even share meals with classmates who hadn’t eaten during hospitalization. While at home, he mainly bought food from convenience stores or ordered takeout, occasionally cooking what he thought were “simple” meals like fried rice. Later, he began preparing more intricate dishes, including stir-fry and stews, and even communicated his culinary outcomes to physicians. Additionally, the patient realized it is crucial to control blood pressure and cholesterol for health as his routine becomes more consistent; those daily life skills are closely associated with maintaining stability in chronic conditions and leading to a positive feedback loop that establishes a strong foundation for the patient’s independent living.

Reintegration into society presents challenges related to social participation, encompassing interactions between patients and others and the independent acquisition of resources necessary for survival. In the inpatient socialization process, patients progressively move from initial behaviors characterized by silence, head avoidance, and continuous mask-wearing to decreasing the use of masks, engaging in active communication, and expressing their feelings and experiences more openly. He can even imitate the words and expressions of others during interactions with group members and initiate brief eye contact with caregivers to seek advice from staff. Learning from higher-functioning patients during group activities increases the probability that low-functioning patients might experience change. This will assist others with comparable experiences and transform and support one another.

Employment serves as a crucial method for acquiring survival resources. Patients with schizophrenia can return to work if they receive systematic therapy and have good prognoses (28), as evidenced by existing studies. Following a year of rehabilitation training, the patient frequently desired employment opportunities. Following an assessment, participation in volunteer work at a day rehabilitation center was recommended, where responsibilities included distributing newspapers and conducting midday rounds. At 14 months: “The work gave me purpose. People said ‘thank you for the newspaper’—small things that made me feel I contribute.” At 24 months: “Every interaction is training for when I work outside. This work enhanced his interaction with peers, healthcare professionals, and other patients’ families while reinforcing and applying the skills acquired through the cycle of ‘learning rehabilitation skills-practicing-encountering problems-relearning in a targeted manner’. This process establishes the groundwork for his reintegration into society and subsequent employment.

#### 4.2.4 Reconciliation and relational repair

Participating in online or in-person family intervention program, the patient’s father gained knowledge about pharmaceutical treatment, symptoms, traits, and other essential medical information, recognizing the essential role of family care and support in the recovery process. His father can keep up steady communication with the case management team to discuss the patient’s condition and any changes.

For the patient himself, there was a noticeable shift in his attitude towards his father. Initially, the patient exhibited resistance and hostility, stating, “I don’t want to see my dad; his tone is just so infuriating”, and “He’s a bad person; he’s not kind at all.” However, through the family intervention program, which focused on improving intimate relationships, the patient gradually began to accept his father. He showed signs of emotional change, such as occasionally calling or saying, “I suddenly miss him. I’m worried about his health.” The patient also shared his complex feelings with the social worker, explaining, “He doesn’t understand this illness,” and “He didn’t know how to handle it, and he wasn’t doing it on purpose.” Over time, the patient was able to let go of his resentment about his father’s inadequate care during the onset of his illness, which had contributed to the worsening of his condition. The improved relationship and the increased family caregiving capacity brought new hope for the patient’s recovery.

#### 4.2.5 From concealment to strategic community engagement

The community serves a supportive function in mitigating the pressure associated with illness for individuals. However, patients frequently experience social isolation and exclusion when reintegrating into the community, which is combined with severe stigma and results in intense resistance. The patient progressively knows the different functions of community hospitals, neighborhood committees, and the Disabled Persons’ Federation, as well as the confidentiality and assistance policies regarding psychopaths, during many conversations with social workers. After 1.5 years of rehabilitation training, patients’ attitudes toward the community shifted. He became more proactive in communicating with community staff about accessible benefits, enquiring about continuity of policy support and engagement in community activities, and stressing privacy to them. The increase in social engagement gives patients a feeling of self-worth and motivation for recovery and pushes them to seek external resources for continued treatment and recovery. Ultimately, this deeper social integration assists individuals in addressing the difficulties of illness. Initially (6 months): “I don’t want community people to know—they’ll treat me differently.” By 30 months: “I told the neighborhood committee about my condition and asked to join activities. They said yes.”

### 4.3 Integration of quantitative and qualitative findings

Quantitative improvements aligned with qualitative narratives. MRSS dependency reduction (29→14) corresponded with the patient’s shift from passive compliance to autonomous daily management. PANSS negative symptom decrease (24→17) paralleled emotional reawakening from numbness to meaningful loneliness. MRSS social interaction improvement (42→19) matched behavioral changes from avoidance to active engagement. The HAMD spike at 12 months was explained through qualitative data showing heightened stigma awareness during community re-engagement—illustrating how mixed methods reveal recovery nuances that scales alone would miss.

## 5 Discussion

About 20–30% of people with schizophrenia have TRS, which unfolds the mental disability because of the incapacity to properly manage the disorder and the severe impairment of social functions (20, 21). There are also restrictions on interventions that fall under the current medical-psychosocial-social framework. Clozapine is acknowledged as an effective treatment for treatment-resistant schizophrenia (TRS), yet it may induce significant side effects. TRS is typically linked to impaired function with verbal expression, attention, memory, comprehension, and family support; the primary challenges in maintaining treatment of rehabilitation are the high cost and the difficulty considering a lack of self-awareness and treatment compliance (29). Therefore, a more all-encompassing approach to TRS rehabilitation must be investigated for accomplishing comprehensive rehabilitation, which encompasses medication therapy, psychosocial interventions, family interventions, and social support services.

### 5.1 The difference between traditional CM from an empowerment perspective

#### 5.1.1 Progress of treatment, objectives, and techniques

Compared to traditional case management that targets to decrease hospitalization rates, relapse frequency, and disease fluctuations, case management for TRS from an empowerment perspective prioritizes patient autonomy and sustainability of recovery through developing self-management skills and reducing relapse risk. This approach aligns with recovery-oriented practice frameworks that emphasize personal agency and self-determination in mental health care (22). To manage the case holistically, the empowerment perspective adopts a multidisciplinary, integrative intervention strategy that involves cooperation between physicians, nurses, rehabilitation specialists, therapists, social workers, and other professionals and advocates for the support of peer groups, community resources and social policies. Personalized recovery plans are developed based on the patient’s unique experiences and requirements. At the same time, the core focus is on ability building, emphasizing restoring their social function and improving life quality. It seeks to continue growth by enhancing their ability to mobilize resources and participate in social activities. This perspective enables the building of new cooperative relationships based on equality and respect through patients expressing their needs and preferences in treatment and participating in planning and decision-making. Research demonstrates that shared decision-making in psychiatric care improves medication adherence and enhances therapeutic alliance (23). Patients escape the conventional hierarchical medical authority dynamic by asserting their rights and moving from passive compliance to active engagement.

#### 5.1.2 Expansion of effect and assessment

Conventional therapy methods concentrate on alleviating symptoms of mental disease, typically using standardized clinical scales to evaluate treatment results according to the frequency and intensity of psychiatric symptoms. However, this may ignore changes in other factors during treatment. Especially patients who lack support are more likely to relapse and make modest gains in quality of life and social functioning. Empowerment interventions are a process-oriented approach that considers the individual goals and needs of the patient. The assessment criteria align with the characteristics of multidisciplinary work. Throughout the empowering process, patients can simultaneously enhance multiple aspects, including disease symptoms, social function, quality of life, social involvement, and dependence reduction, which can help patients manage stress and obstacles more effectively.

#### 5.1.3 Diversity of themes and subjects

Hospitals, mental health institutions, and communities are primarily settings for traditional case management, with a disconnect between professional disease management and community or family engagement. Although the inpatient day rehabilitation center is the central location of empowerment case management for TRS, patients can select their admission time and duration. By fostering a supportive environment, open, flexible group sessions are conducted to guide patients toward self-directed involvement. Furthermore, day rehabilitation centers also emphasize the involvement of social networks and family members in order to replicate real-life contexts. As the core subject of rehabilitation, empowerment case management links a network of relationships for them, in which various roles play different and crucial roles in facilitating recovery.

### 5.2 Comparison with existing TRS treatment evidence

Naturalistic studies of TRS patients on optimized pharmacotherapy alone typically report persistent functional impairment despite partial symptom stabilization. Meta-analyses of psychosocial interventions for TRS indicate that cognitive remediation produces moderate effect sizes for cognitive functioning but limited functional gains (24), while family psychoeducation improves medication adherence and reduces relapse but shows variable effects on social functioning (25). Our case demonstrated improvements across symptom, functional, and family domains concurrently, suggesting that integrated multidimensional approaches combining elements of medication management training, cognitive-behavioral strategies, social skills training, and family intervention may produce more comprehensive outcomes than single-component interventions. However, controlled comparisons are necessary to confirm this hypothesis.

### 5.3 Addressing TRS-specific challenges

This model addresses TRS barriers through cognitive compensation strategies (visual medication aids, repetitive training, environmental cues for executive dysfunction), motivation-building mechanisms (immediate tangible rewards, peer modeling for negative symptoms), and reframing adherence as active symptom management rather than passive compliance. Standard case management typically emphasizes service linkage without these neurocognitive adaptations.

### 5.4 Mechanisms and critical components

Qualitative analysis suggests pathological treatment empowerment (medication self-management and symptom monitoring) provided a stabilizing foundation enabling engagement with other intervention components. Social participation empowerment showed the most substantial quantitative improvement (MRSS social interaction subscale decreased from 42 to 19), potentially because skills training provided concrete tools immediately applicable to daily life. The patient’s subjective experience reflected a progressive shift from viewing himself as a passive recipient of care to an active agent in recovery, stating in later interviews that rehabilitation training helped him ‘take control’ and ‘not just rely on doctors.’ However, the interaction between empowerment dimensions cannot be fully disentangled from this single case. Component analysis through dismantling designs in future controlled studies would clarify which elements are necessary versus sufficient for producing observed outcomes.

### 5.5 Cultural context and implementation considerations

This intervention was implemented within Chinese cultural and healthcare contexts, which may influence empowerment processes and implementation feasibility. Collectivist values emphasizing family interdependence and filial responsibility align naturally with the strong family intervention component in our model, potentially enhancing engagement compared to more individualistic frameworks. However, stigma operates distinctively in contexts prioritizing “saving face”, making community reintegration particularly challenging for patients with visible psychiatric histories. The patient’s gradual shift from avoidance (‘I simply ignore neighbors’) to proactive engagement (‘I can try to make friends with them’) suggests that structured empowerment interventions may help navigate stigma-related barriers specific to Chinese social contexts. Additionally, China’s developing community mental health infrastructure, with day rehabilitation centers embedded within specialized psychiatric hospitals rather than community settings, creates unique implementation opportunities and constraints that may affect generalizability to other healthcare systems.

### 5.6 The advantages of the empowerment perspective

Empowerment links people’s positive actions and support systems to the community and society, making patients and caregivers consider strengths and weaknesses, abilities and limitations, as well as health and illness. The emergence of this strategy reflects a growing focus on patient-centered humanistic care in mental health, giving patients opportunities to develop knowledge and skills in cooperative partnerships between patients, caregivers and professionals.

#### 5.6.1 Maintain stability of disease treatment

The approach may decrease the possibility of acute episodes and symptom exacerbation because patients can recognize early signs of relapse and take appropriate actions promptly under the train of monitoring symptoms and managing medications. Early warning signs monitoring has been shown to reduce relapse rates in schizophrenia by enabling timely intervention (26). When they comprehend the equal value of subjective agency and medication in treatment, it can trigger intrinsic motivation for recovery and lead them to engage more actively in the self-management of their illness. During the dynamic capacity-building process, patients receive support to reconstruct their sense of autonomy and subjectivity to adapt to challenges and changes encountered at various stages of treatment and rehabilitation.

#### 5.6.2 Bring patients to the center

The model promotes patients to become energetic propellent and enforcers when involved in decision-making regarding treatment strategies, rehabilitation objectives, and medication usage. Through coordination and multi-party communication, patients can clarify their desires and emotions due to improved social skills and self-awareness, which addresses the limitations of conventional treatments that entail post-schizophrenia depression, fear, and loss of belief. All of this supports long-term mental health for convalescent persons.

#### 5.6.3 Integrate resources for patients

People with TRS have a deterioration in social function, which is a societal issue as well as a medical one. Empowerment underscores patients’ assumption of social roles and integration into society through rehabilitation plans tailored to their interests, abilities, and life objectives, including educational program, vocational training, social skills development, and supportive environments. For long-term rehabilitative patients, empowerment promotes a cooperative model that incorporates resources to offer comprehensive support, creating a secure and peaceful living environment to facilitate their effective transition from inpatient to independent community living. As patients exhibit better self-management abilities and social function, their social integration progressively modifies the stigma and stereotype of schizophrenia patients as incompetent or uncontrollable. Community-based interventions that enhance social participation have been shown to reduce internalized stigma and improve self-esteem in people with serious mental illness (27), which ultimately reshapes a virtuous cycle because of more acceptance of this group.

### 5.7 Feasibility and scalability

Core elements include medication self-management training, monthly multidisciplinary communication, and family psychoeducation. Adaptable components for resource-limited settings include session frequency (weekly vs. twice-weekly), team size (nurse coordinator facilitating fewer specialists), and delivery mode (community-based or technology-assisted). The primary constraint is clinician training in collaborative rather than directive approaches.

### 5.8 Genetic burden and treatment implications

This case presents exceptional genetic loading with three generations of affected relatives and maternal suicide. Despite this severe family history of schizophrenia, the patient showed meaningful improvement with intensive intervention, suggesting that comprehensive psychosocial rehabilitation may benefit even in the presence of substantial genetic vulnerability. The traumatic loss of his mother to suicide was addressed through the psychotherapy component, though the specific impact of this grief work on overall outcomes was not systematically evaluated. Future research should examine whether family history moderates response to empowerment-based interventions.

### 5.9 Limitations

This case report has several important limitations. First, as a single case study, the findings cannot be generalized to broader TRS populations. The observed improvements may reflect the natural course of illness, placebo effects, or other unmeasured factors rather than the intervention itself. Second, the absence of a control condition precludes causal inferences about treatment effectiveness. Third, the patient’s characteristics—including relatively preserved baseline functioning, paternal support, and ability to attend day rehabilitation—may not represent typical TRS patients, particularly those with severe negative symptoms or lacking family support. Fourth, all outcome assessments were conducted by the treating psychiatrist without blinded evaluation, introducing potential detection bias that may have inflated observed improvements. Fifth, although medication remained stable throughout the 3-year intervention period, the synergistic effects of pharmacological and psychosocial treatments cannot be fully disentangled without a control condition. Sixth, the resource intensity of this approach (3-year duration, multidisciplinary team, twice-weekly sessions) raises feasibility concerns for routine implementation. Finally, the cultural context of Beijing’s mental health infrastructure may limit transferability to other settings. These limitations underscore that this report should be viewed as hypothesis-generating rather than hypothesis-testing.

## 6 Relevance to practice

Adopting an empowerment-oriented case management model for individuals with treatment-resistant mental disorders may facilitate a shift from compliance-focused care to collaborative, person-centered recovery. Recognizing patients as active agents in their own rehabilitation—rather than passive recipients of care—can support the development of tailored strategies that align with their lived experiences, coping capacities, and recovery goals. This approach also underscores the importance of building sustained therapeutic relationships and flexible care plans responsive to patients’ fluctuating needs. Engaging patients in shared decision-making and reflective dialogue about their illness narratives, treatment preferences, and social reintegration challenges may enhance their sense of agency and promote trust. Furthermore, involving multidisciplinary teams in reflective practice and training on empowerment principles may help foster a more respectful, strengths-based clinical culture. Such practice relevance not only expands the therapeutic space for treatment-resistant populations, but also invites a reconsideration of how clinical knowledge is co-produced with patients in complex psychiatric contexts.

## Data Availability

The raw data supporting the conclusions of this article will be made available by the authors, without undue reservation.
